# Vascular responses to radiotherapy and androgen-deprivation therapy in experimental prostate cancer

**DOI:** 10.1186/1748-717X-7-75

**Published:** 2012-05-23

**Authors:** Kathrine Røe, Lars TG Mikalsen, Albert J van der Kogel, Johan Bussink, Heidi Lyng, Anne H Ree, Laure Marignol, Dag R Olsen

**Affiliations:** 1 Department of Radiation Biology, Institute for Cancer Research, Oslo University Hospital, PO Box 4953 Nydalen, 0424 Oslo, Norway; 2 Institute of Clinical Medicine, University of Oslo, Oslo, Norway; 3 Department of Oncology, Akershus University Hospital, Lorenskog, Norway; 4 Department of Physics, University of Oslo, Oslo, Norway; 5 Department of Radiation Oncology, Radboud University Nijmegen Medical Center, Nijmegen, The Netherlands; 6 Department of Human Oncology, University of Wisconsin, Madison, WI, USA; 7 Prostate Molecular Oncology Research Group, Academic Unit of Clinical and Molecular Oncology, Institute of Molecular Medicine, St. James’s Hospital and Trinity College Dublin, Dublin, Ireland; 8 Faculty of Mathematics and Natural Sciences, University of Bergen, Bergen, Norway

**Keywords:** Prostate cancer, Androgen-deprivation therapy, Radiotherapy, Immunohistochemistry, Dynamic contrast-enhanced MRI

## Abstract

**Background:**

Radiotherapy (RT) and androgen-deprivation therapy (ADT) are standard treatments for advanced prostate cancer (PC). Tumor vascularization is recognized as an important physiological feature likely to impact on both RT and ADT response, and this study therefore aimed to characterize the vascular responses to RT and ADT in experimental PC.

**Methods:**

Using mice implanted with CWR22 PC xenografts, vascular responses to RT and ADT by castration were visualized in vivo by DCE MRI, before contrast-enhancement curves were analyzed both semi-quantitatively and by pharmacokinetic modeling. Extracted image parameters were correlated to the results from ex vivo quantitative fluorescent immunohistochemical analysis (qIHC) of tumor vascularization (9 F1), perfusion (Hoechst 33342), and hypoxia (pimonidazole), performed on tissue sections made from tumors excised directly after DCE MRI.

**Results:**

Compared to untreated (Ctrl) tumors, an improved and highly functional vascularization was detected in androgen-deprived (AD) tumors, reflected by increases in DCE MRI parameters and by increased number of vessels (*VN*), vessel density ( *VD*), and vessel area fraction ( *VF*) from qIHC. Although total hypoxic fractions ( *HF*) did not change, estimated acute hypoxia scores ( *AHS*) – the proportion of hypoxia staining within 50 μm from perfusion staining – were increased in AD tumors compared to in Ctrl tumors. Five to six months after ADT renewed castration-resistant (CR) tumor growth appeared with an even further enhanced tumor vascularization. Compared to the large vascular changes induced by ADT, RT induced minor vascular changes. Correlating DCE MRI and qIHC parameters unveiled the semi-quantitative parameters area under curve ( *AUC*) from initial time-points to strongly correlate with *VD* and *VF*, whereas estimation of vessel size ( *VS*) by DCE MRI required pharmacokinetic modeling. *HF* was not correlated to any DCE MRI parameter, however, *AHS* may be estimated after pharmacokinetic modeling. Interestingly, such modeling also detected tumor necrosis very strongly.

**Conclusions:**

DCE MRI reliably allows non-invasive assessment of tumors’ vascular function. The findings of increased tumor vascularization after ADT encourage further studies into whether these changes are beneficial for combined RT, or if treatment with anti-angiogenic therapy may be a strategy to improve the therapeutic efficacy of ADT in advanced PC.

## Background

Androgens play key regulatory functions in prostate cancer (PC) growth and proliferation. Androgen-deprivation therapy (ADT) is established as an integral component of contemporary management of advanced PC, providing reduction of androgen-sensitive tumor cells and symptomatic relief for the patients [1]. However, the lack of durable remission indicates that current ADT strategies are suboptimal, or that successful treatment outcome may require ADT to be combined with other therapeutic interventions. Specifically, the combination of ADT with radiotherapy (RT) has proven efficacious [2,3], yet the optimal timing of RT in relation to ADT remains unclear.

Tumor vascularization is an important physiological feature of tumor tissue, influencing both RT and ADT response. Adequate tumor oxygenation results in enhanced radical formation following RT, inducing irreversible DNA damage and subsequent tumor cell death [4]. Well-vascularized tumors are more likely to be well oxygenated, in contrast to poorly vascularized tumors often suffering from tumor hypoxia, reducing the efficacy of combined treatments, including RT. Moreover, ADT affects tumor vascularization by its direct influence on androgen-sensitive, endothelial cells. Although the endothelial cells express androgen receptors providing paracrine signaling and vascular support facilitating recurrent growth, the role of androgens in regulation of angiogenesis is still not fully understood [5,6]. Further, most investigations have focused on the initial vascular response following ADT, typically from a few hours up to one week. The more long-term effects of ADT on tumor vasculature may be equally or more important in understanding PC progression, and for selection of combined treatments.

Quantitative dynamic contrast-enhanced magnetic resonance imaging (DCE MRI) is a promising non-invasive imaging modality for assessing tumor vacularization and treatment responses [7]. Conventional extracellular contrast agents rapidly distribute throughout the blood plasma and extravasate into the extracellular extravascular space (EES) after being intravenously (i.v.) injected. These agents affect the tissues’ relaxation rates, causing increased signal intensity on T1-weighted MR images. The signal intensity changes versus time reflect vascular features and may be analyzed directly using semi-quantitative approaches or quantitatively by pharmacokinetic models. Extracted image parameters enable estimation of the tumors’ vascular characteristics and their angiogenic responses to therapeutic interventions, potentially also serving as indirect non-invasive biomarkers of tumor hypoxia. However, the use of DCE MRI parameters is dependent on correct tumor physiological interpretations. The current understanding is still incomplete and to a large extent based on assumptions yet to be validated. Therefore, the present study also interpreted how the DCE MRI parameters we quantified correlated to the underlying biology, by performing a quantitative analysis of fluorescent immunohistochemistry (qIHC) of excised tumor tissue.

Thus, this study aimed to characterize the vascular responses to RT and ADT using an experimental, androgen-sensitive PC model. Animals were subjected to in vivo DCE MRI and analyzed with both semi-quantitatively and by pharmacokinetic models. The animals’ tumors were excised immediately after MRI for quantitative ex vivo analysis of tumor vascularization (9 F1), perfusion (Hoechst 33342), and hypoxia (pimonidazole).

## Materials and methods

### Experimental tumor model

Tumors were generated by subcutaneous (s.c.) implantation of (~2 mm)^3^ tumor tissue from the human, androgen-sensitive CWR22 PC xenograft, together with a 12.5 mg sustained-release testosterone pellet (Innovative Research of America, Sarasota, FL), into adult male Balb/c nude mice. These pellets ensure stable, human serum testosterone levels and contribute to improved recapitulation of androgen-regulated homeostasis [8]. Animal experiments were performed in accordance with protocols approved by the institutional animal care and use committee, in compliance with guidelines on animal welfare of the Norwegian National Committee for Animal Experiments. Tumor sizes were measured using calipers from implantation until the end of the experiment.

### Treatments and experimental groups

The study included two consecutive experiments with untreated (Ctrl), irradiated (RT), androgen-deprived (AD), and castration-resistant (CR) tumors. The CWR22 xenograft mimicks the clinical situation of PC disease progression, by regressing after ADT, and showing stability during long-term ADT before recurrent CR tumor growth is evident, a disease which presently is incurable in the clinic. To allow tumor size-independent comparisons all analyzed tumors had diameters of 8 mm.

Firstly, animals in the Ctrl group (n = 4) were subjected to DCE MRI when the tumor diameter reached 8 mm. For the RT group (n = 6), tumors reaching 8 mm in diameter were irradiated with a single dose of 15 Gy, followed by DCE MRI 24 h later, a time-point selected based on a previous study investigating radiation-induced effects in the CWR22 xenograft [9]. Radiation was delivered using a ^60^Co source (Mobaltron 80, TEM Instruments, Crawley, UK), with a dose rate of 0.8 Gy/min. Tumor irradiation was performed by placing the tumors in the corners of a 10 × 10 cm radiation field, keeping the animals’ bodies outside the field to minimize radiation dose deposition to normal tissues. ADT was performed when the shortest tumor diameter reached 12 mm, by surgically castrating the animals and removing the testosterone pellet. One month post-castration the tumor size was reduced to 8 mm in diameter, upon which the animals were subjected to DCE MRI (n = 4). Following MRI all tumors were excised and immediately snap-frozen for qIHC.

Secondly, the experiment was repeated to analyze Ctrl (n = 5), AD (n = 5), and CR (n = 5) tumors of identical size as in the first experiment. All tumors were subjected to DCE MRI, and blood sampling was performed for measuring serum prostate-specific antigen (PSA) levels.

Radiation, castration, and MRI were performed under anesthesia, after s.c. injections of a mixture of 2.4 mg/ml tiletamine and 2.4 mg/ml zolazepam (Zoletil vet, Virbac Laboratories, Carros, France), 3.8 mg/ml xylazine (Narcoxyl vet, Roche, Basel, Switzerland), and 0.1 mg/ml butorphanol (Torbugesic, Fort Dodge Laboratories, Fort Dodge, IA), diluted 1:5 in sterile water, in doses of 5 μl/g prior to radiation, and 7.5 μl/g before MRI acquisitions and castration. Analgesia was provided to castrated animals by s.c. injections of buprenorphine (Temgesic, Schering-Plough, Brussels, Belgium) in a dose of 0.1 mg/kg.

### Prostate-specific antigen

Blood samples were withdrawn from the animals’ femoral veins immediately before euthanasia and tumor excision. The samples coagulated before being centrifuged and frozen at −80°C until all samples were analyzed simultaneously. Serum PSA levels were measured using the fluoroimmunometric AutoDELFIA ProStatus PSA Free/Total kit (PerkinElmer Life and Analytical Sciences, Wallac Oy, Turku, Finland).

### DCE MRI acquisition and analysis

**Figure 1:**
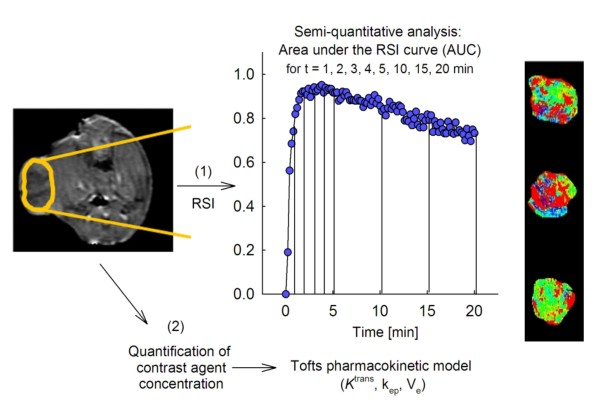
**Dynamic contrast-enhanced MRI of experimental prostate cancer.** Following dynamic contrast-enhanced (DCE) MRI the tumors were traced in the T_1_-weighted images. Firstly, semi-quantitative analysis was performed on the time-dependent relative signal intensity (*RSI(t))* curves, by calculating the area under the *RSI* curve *(AUC(t))*, of each tumor voxel, for different time-points. Secondly, pharmacokinetic analysis was performed using the Tofts model, producing parametric tumor maps of *K*^trans^, *k*_*ep*_, and *V*_*e*_. Examples of parametric tumor maps are shown to the right.

DCE MRI was acquired as previously described [10], and analyzed using in-house developed software in IDL (Interactive Data Language v6.2, Research Systems Inc., Boulder, CO), as illustrated in Figure 1. For the central slice of each tumor, a region-of-interest (ROI) was manually traced in T_1_-weighted DCE MR images, excluding surrounding skin and connective tissue. Firstly, semi-quantitative analysis was performed. The time-dependent relative signal intensity (*RSI(t)*) was calculated for each image voxel using the equation *RSI(t) = (SI(t) – SI(0))/SI(0),* where *SI(0)* refers to the pre-contrast signal intensity and *SI(t)* the post-contrast signal intensity in the voxel at time *t*. The semi-quantitative parameter area under the *RSI(t)* curve ( *AUC(t))* was calculated for *t* = 1, 2, 3, 4, 5, 10, 15, and 20 min.

Secondly, pharmacokinetic analysis was performed using the two-compartment Tofts model [11]. Signal intensities were converted into contrast agent (CA) concentration (*C(t))* by using the method of Hittmair [12]. The vascular input function (VIF) needed in Tofts modeling has previously been established for our male mouse strain: VIF = 3.57 e^-(0.025)t^ + 1.45 e^-(0.0074)t^[10]. Using the VIF parameters and the CA concentration curves, each voxel was fitted to the Tofts model, allowing estimations of the forward volume transfer constant of CA from blood plasma into the EES, *K*^trans^ (s^-1^), the backward transfer constant from EES to blood plasma, *k*_*ep*_ (s^-1^), and the fractional distribution volume of CA, *V*_*e*_ (a. u.), as derived from *K*^trans^/*k*_*ep*_. When performing Tofts modeling, the fit fails to converge for some voxels due to low CA uptake or voxels providing negative values. These unfitted voxels were set equal to 0 and included in calculation of parametric tumor maps. The unfit fraction; i.e. the sum of unfitted voxels versus the total number of voxels within the tumor ROI, was monitored to investigate the possible relation between this fraction and tumor necrosis assessed in the tissue sections.

### Immunohistochemistry

Sixty minutes prior to euthanasia, 80 mg/kg of pimonidazole hydrochloride (1-[(2-hydroxy-3-piperidinyl)propyl]-2-nitroimidazole hydrochloride, Natural Pharmacia International Inc., Burlington, MA, USA) in 0.5 ml saline was injected intraperitoneally (i.p.) in the animals to determine tumor hypoxia. Tumor perfusion was found by i.v. injection of 15 mg/kg Hoechst 33342 (Sigma-Aldrich, Oslo, Norway) in 0.1 ml saline 5 min before euthanasia. Immediately after dissection, tumors were snap-frozen in liquid nitrogen and stored at −80°C until being shipped to the Department of Radiation Oncology, Radboud University, Nijmegen Medical Center, Nijmegen, The Netherlands, for staining and imaging. The central slice of the tumor was cut before being stained and scanned according to previously described procedures [13], to obtain three spatially matched immunofluorescent images of tumor vascularization (9 F1; TCL image, TNO, Delft, The Netherlands), tissue perfusion (Hoechst 33342), and hypoxia (pimonidazole).

### Quantitative analysis of immunohistochemistry

Spatial maps of hypoxia, vessels, and perfused area were created from immunofluorescent images. Firstly, hypoxic regions were determined from pimonidazole stains using MATLAB 7.0.1 (The MathWorks, Natick, MA). Different methods were used due to differences in signal intensity and image contrast between different batches. For nine cases: (a) the image was digitally filtered to correct for unequal illumination in each tile, (b) a Gaussian blur was applied to smooth the image (σ = 8 px), and (c) the threshold was set to the mean of the 5^th^ and 95^th^ percentile intensity value. For six cases the threshold was set to 0.3 × 95^th^ intensity percentile, and in one case the threshold was set manually. Secondly, vessels were manually delineated in 9 F1 stains. Thirdly, perfusion was manually segmented using local and/or global thresholds in Hoechst stains. The following regions were manually delineated based on a combination of stains: (a) *gross tumor* region, (b) *artifacts*, (c) *necrosis*; regions with a strong background signal from 9 F1 and a low signal from pimonidazole, (d) *other tissue*; tumor regions with markedly different texture in 9 F1 and pimonidazole stains, and a defined transition to viable tumor regions. Two additional tumor regions were defined: (e) *net tumor*; gross tumor excluding artifacts, and (f) *viable tumor*; net tumor excluding necrosis and other tissue. The necrotic fraction ( *NF*) was defined as necrotic area/net tumor area.

A vessel was defined as an independent contiguous 8-connected region in the vessel map, and lumens were considered part of the vessel. The following vessel parameters were extracted: total number of vessels (*VN*), vessel density ( *VD)* as *VN*/viable tumor area, vascular area fraction ( *VF*) as vascular area/viable tumor area, and mean vessel size ( *VS*).

A vessel was considered to be perfused if it was less than 20 μm away from a perfusion stain with at least 10% of its area. The following perfusion parameters were extracted: perfusion stain fraction (*PF*) as perfusion stain area/viable tumor area, and perfused vessel fraction ( *PVF*) as number of perfused vessels/ *VN*.

These parameters measured the degree of hypoxia: hypoxic fraction (*HF)* as hypoxic area/viable tumor area, and mean distance from hypoxic pixels to nearest vessel ( *DHV*). Since pimonidazole staining intensity could not be converted to oxygen pressure (pO_2_), the exact boundary between hypoxia and normoxia cannot be decided; *HF* and *DHV* are considered to be estimates proportional to true values. The theoretical normoxic fraction was calculated based on the assumption that all (perfused) vessels supply oxygen and that the diffusion range of oxygen is 70 μm: fraction of viable tumor tissue < 70 μm away from a vessel ( *VF70V*).

Estimation of acute hypoxia was based on the following assumptions: (a) since transport of Hoechst requires a functional and oxygenated vascular network, areas with perfusion staining were considered functional after Hoechst injection; (b) areas of hypoxia staining were considered to reflect hypoxia after pimonidazole injection. By assuming that the metabolic activity was sufficiently low to not affect the diffusion distance of oxygen, it was further assumed that the diffusion distance of oxygen was longer than the diffusion distance of Hoechst, and thus, that the amount of pimonidazole staining within a 50 μm distance from perfusion stains was proportional to the amount of acute hypoxia. The proportion of the total *HF* that was acute was named “acute hypoxia score” ( *AHS*). The remaining proportion is thus representing chronic hypoxia (= 1 – *AHS)*.

The manual delineations and thresholds were performed in Gimp 2.6.4 (http://www.gimp.org), and quantitative analysis in an in-house program written in Python 2.6.6 (http://www.python.org), with the scipy extension (http://www.scipy.org).

### Statistics

Statistical analysis was performed using PASW Statistics 18.0 (IBM, Somers, NY). Differences were analyzed using two-sided *t*-tests and correlations assessed by Pearson’s correlations ( *r*). A significance level of 5% was used.

## Results

### Assessment of response to radiotherapy and androgen-deprivation therapy by quantitative immunohistochemistry

**Figure 2:**
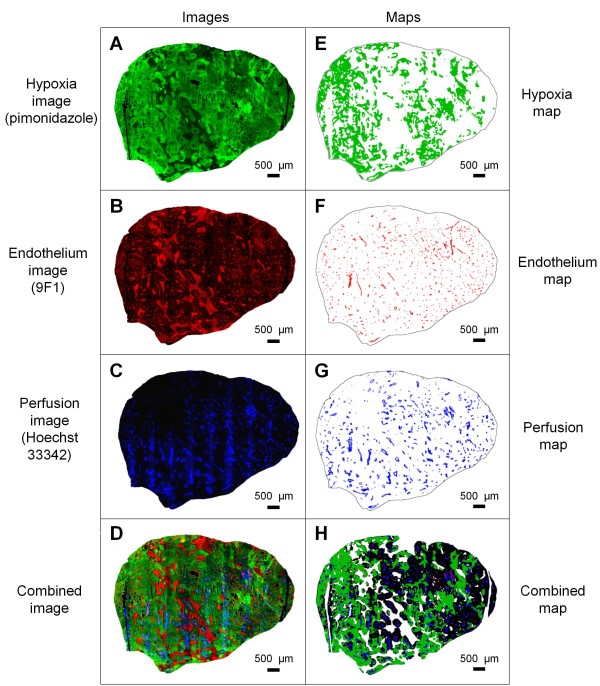
**Fluorescent immunohistochemical images and maps.** Tumors were triple-stained with fluorescent immunohistochemical markers of hypoxia (pimonidazole), endothelium (9 F1), and perfusion (Hoechst 33342), to produce images of hypoxia ( **A**), endothelium ( **B**), perfusion ( **C**), and a combined image ( **D**). Corresponding maps were extracted and used in the quantitative analysis ( **E-G**). Composite map where artefacts and necrosis, which were excluded from the measurements, are shown in white ( **H**).

To characterize the response to RT and ADT, qIHC was performed. Matching sets of images of tumor hypoxia (pimonidazole), endothelium (9 F1), and perfusion (Hoechst 33342) (Figures 2A–C), as well as a combined image (Figure 2D), were obtained. Corresponding maps of the tumor-specific features (Figures 2E–H) were extracted and used in qIHC.

**Figure 3:**
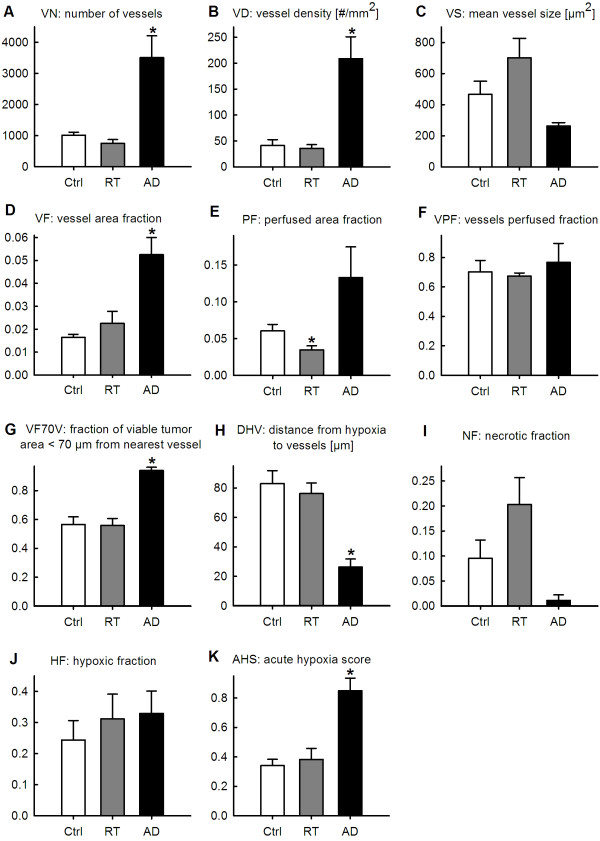
**Quantitative immunohistochemical analysis.** Intergroup differences in parameters from quantitative immunohistochemical analysis of triple-stained tumor tissue sections (9 F1; endothelium, Hoechst 33342; perfusion, pimonidazole; hypoxia) from untreated (Ctrl), irradiated (RT), and androgen-deprived (AD) prostate carcinoma (PC) xenografts, presented as mean and s.e.m. per group. Significant differences ( *p* < 0.05) compared to Ctrl tumors are indicated with a *.

Figure 3 shows the results summarized per experimental group. Compared to Ctrl tumors, AD tumors presented significant increases in several vascular qIHC parameters; a 3.5-fold increase in *VN* ( *p* = 0.012) (Figure 3A), a 5.1-fold increase in *VD* ( *p* = 0.009) (Figure 3B), and a 3.2-fold increase in *VF* ( *p* = 0.003) (Figure 3D). The viable tissue was located closer to the vasculature in AD tumors compared to in Ctrl tumors, as reflected by a 1.7-fold increase in *VF70V* ( *p* < 0.001) (Figure 3G). Also the hypoxic stains were closer to vessels in AD tumors, reflected by *DHV* being 0.7-fold decreased ( *p* = 0.002) (Figure 3H) compared to Ctrl tumors. AD induced an insignificant decrease in *VS* (Figure 3C) and *NF* (Figure 3I). The changes in *PF* (Figure 3E) and *VPF* (Figure 3F) in AD tumors compared to Ctrl tumors were statistically insignificant; yet they indicate that tumors regressing in size after ADT acquire more vessels at a higher density, and remain equally perfused and highly functional.

Following RT, the sole significant vascular change compared to Ctrl tumors was the 0.4-fold decrease in *PF* ( *p* = 0.031) (Figure 3E). The changes in *VN* (Figure 3A), *VD* (Figure 3B), *VS* (Figure 3C), *VF* (Figure 3D), *DHV* (Figure 3H), and *NF* (Figure 3I) were insignificant. The other parameters (*VPF*, *VF70V*) remained unchanged.

**Figure 4:**
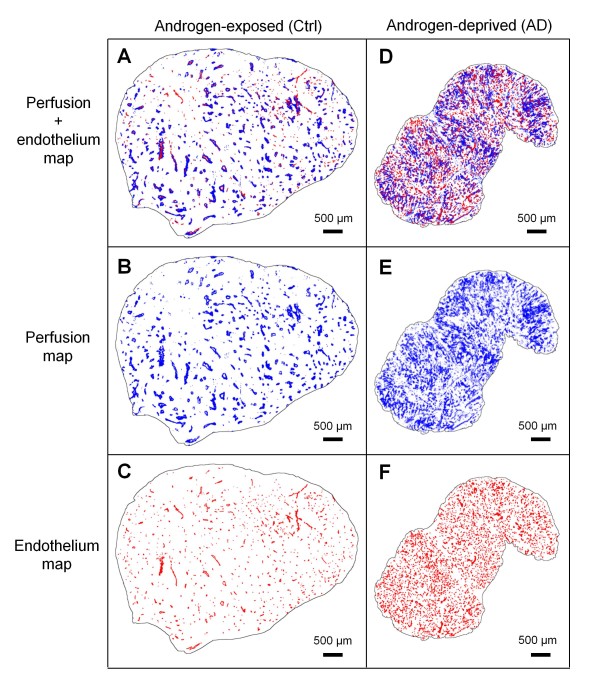
**Tumor vascularization in androgen-deprived versus untreated tumors.** Increased tumor vascularization in an androgen-deprived (AD) tumor (left column) compared to untreated (Ctrl) tumor (right column) visualized by combined perfusion and endothelium maps ( **A** and **D**, respectively), and separate maps of perfusion ( **B** and **E**, respectively) and endothelium ( **C** and **F**, respectively).

Generally, these results unveiled minor radiation-induced effects on tumor vasculature, whereas AD tumors showed significantly increased tumor vascularization. Compared to the Ctrl cohort, AD tumors developed many small vessels covering a significantly larger fraction of the total tumor area. This increased tumor vascularization amongst AD tumors is visualized by perfusion and endothelium maps in Figure 4.

The *HF* did not differ significantly between the groups (Figure 3J). However, independent of *HF* the estimated *AHS* was 2.5-fold higher ( *p* = 0.002) in AD tumors compared to in Ctrl tumors, whereas RT tumors only showed an insignificant increase (Figure 3K).

### Assessment of response to radiotherapy and androgen-deprivation therapy by dynamic contrast-enhanced MRI

**Figure 5:**
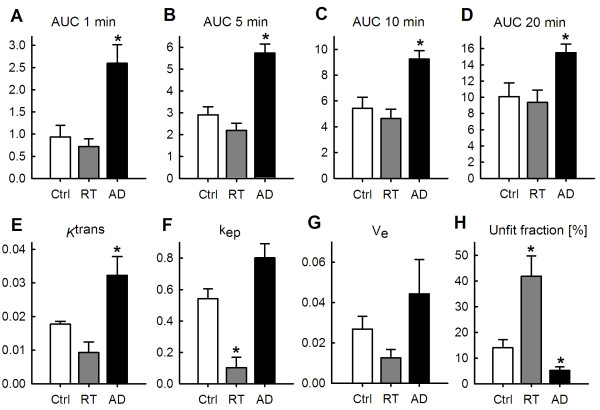
**Semi-quantitative and pharmacokinetic analysis of dynamic contrast-enhanced MRI.** Both semi-quantitative and pharmacokinetic DCE MRI parameters unveiled increased vascularization in androgen-deprived (AD) tumors. Intergroup differences between untreated (Ctrl), irradiated (RT), and androgen-deprived (AD) prostate carcinoma (PC) xenografts for the semi-quantitative parameter area under the relative signal intensity curve ( *AUC)* (a.u.) for 1 min ( **A**), 5 min ( **B**), 10 min ( **C**), and 20 min ( **D**), and Tofts kinetic model parameters *K*^trans^ (s^-1^) (E), *k*_*ep*_ (s^-1^) (F), and *V*_*e*_ (a.u.) (G), in addition to the fraction of unfitted voxels (H), summarized as mean and s.e.m. per group. Significant differences (*p* < 0.05) compared to Ctrl tumors are indicated with a *.

Figures 5A-D show that inter-group differences in *AUC* values were most evident at initial time-points and less pronounced at later time-points. Compared to Ctrl tumors, RT induced insignificant changes in *AUC* values, whereas AD tumors presented significantly higher *AUC* values than Ctrl tumors at all analyzed time-points, with the largest increase at the initial time-point, *AUC 1 min*, being 2.8-fold (p = 0.016). The *AUC* values remained high in AD tumors during the initial time-points before gradually decreasing relative to Ctrl tumors, the difference being 2.5-fold at 2 min ( *p* = 0.007), 2.3-fold at 3 min ( *p* = 0.004), 2.0-fold at 4 min ( *p* = 0.002), 2.0-fold at 5 min ( *p* = 0.002), 1.7-fold at 10 min ( *p* = 0.012), 1.6-fold at 15 min ( *p* = 0.020) and 1.5-fold at 20 min ( *p* = 0.034).

In Figures 5E-G, intergroup differences in Tofts pharmacokinetic parameters are shown. Compared to Ctrl tumors, RT induced a 0.5-fold reduction in *K*^trans^, whereas a significant 1.8-fold increase was unveiled in AD tumors (*p* = 0.041) (Figure 5E). For *k*_*ep*_, RT tumors showed a 0.8-fold reduction (*p* = 0.002), in contrast to a 1.5-fold increase in AD tumors, when compared to Ctrl tumors (Figure 5F). Large intra-group differences in *V*_*e*_ resulted in insignificant changes in both RT and AD tumors (Figure 5G). The unfit fraction showed a 3.0-fold increase (*p* = 0.026) in RT tumors, and a 0.6-fold reduction ( *p* = 0.046) in AD tumors, compared to Ctrl tumors (Figure 5H).

### Correlations between quantitative immunohistochemistry and dynamic contrast-enhanced MRI

**Figure 6:**
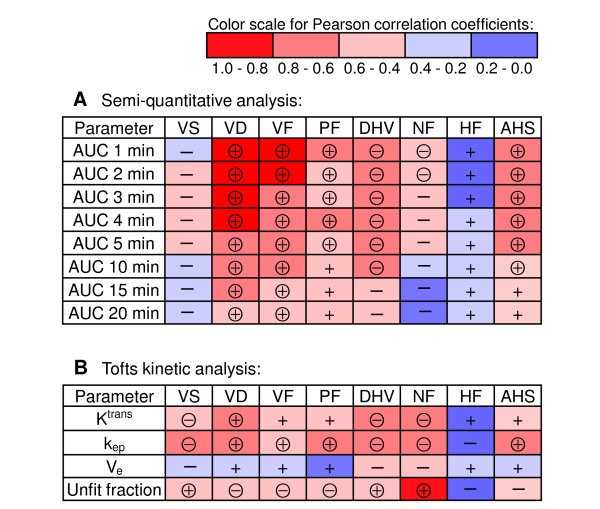
**Correlations between parameters from dynamic contrast-enhanced MRI and quantitative immunohistochemistry.** Correlations between DCE MRI parameters from ( **A**) semi-quantitative and ( **B**) Tofts kinetic analysis, and qIHC parameters. Color labeling reflects correlation strength (Pearson correlation coefficients), as indicated in the color scale (top). Positive and negative correlations are indicated with + and –, respectively, and encircled + and – denotes significant correlations ( *p* < 0.05). Abbreviations: *AUC*; area under the relative signal intensity ( *RSI*) curve, *VS*; mean vessel size (μm^2^), *VD*; vessel density (number of vessels/mm^2^), *VF*; vessel area fraction, *PF;* perfused area fraction, *DHV*; distance from hypoxia to vessels (μm), *NF*; necrotic fraction, *HF*; hypoxic fraction, *AHS*; acute hypoxia score\.

Figure 6 shows resulting Pearson correlation coefficients (*r*) from correlating parameters from DCE MRI and qIHC. Initial *AUC* values (< 4 min) correlated very strongly and positively ( *r =* 0.8–1.0) to *VD* and *VF*, whereas correlations at later time-points were less pronounced ( *r* = 0.6–0.8). The Tofts parameters *K*^trans^ and *k*_*ep*_ also correlated significantly to *VD*, although not as strong as the *AUC* values ( *r* = 0.6–0.8). Tumor perfusion, as reflected by *PF*, was weakly correlated to *AUC* values ( *r* = 0.4–0.6). Of note, *VS* correlated only insignificantly, and negatively, to all *AUC* values. *NF* was negatively correlated to initial *AUC* values ( *r* = 0.4–0.6). The overall *HF* did not correlate to any *AUC* parameter, but *DHV* correlated negatively ( *r =* 0.6–0.8) to all *AUC* values obtained from 1 min to 10 min. The *AHS* fraction correlated positively and very strongly to *NF* ( *r* = 0.85). Tofts parameters correlated poorly to *HF*, but *k*_*ep*_ was strongly positively correlated to *AHS* (r = 0.72).

### Vascular changes occurring in the development of castration-resistant disease

**Figure 7:**
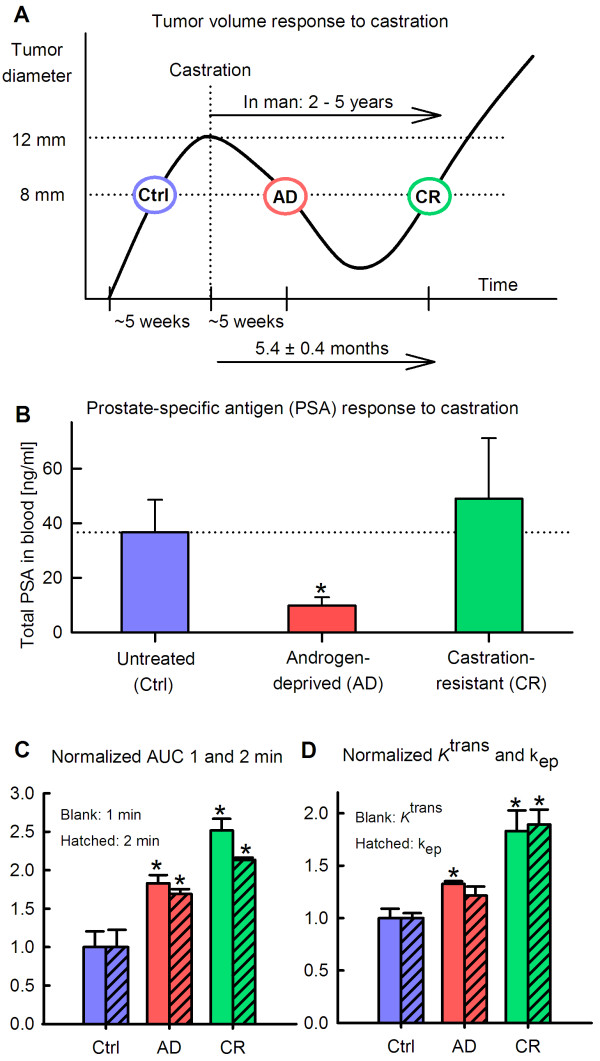
**Vascular changes in the transition from untreated (Ctrl), via androgen-deprived (AD), to castration-resistant (CR) disease.** Response to androgen-deprivation therapy (ADT) on tumor volumes, showing that CR tumors developed 5.4 ± 0.4 months after ADT ( **A**). Response to ADT on serum prostate-specific antigen (PSA) expression measured in blood samples withdrawn from the animals ( **B**). The vascular parameters *AUC 1 min* and *AUC 2 min* calculated from semi-quantitative analysis of DCE MRI ( **C**). The vascular parameters *K*^trans^ and *k*_*ep*_ derived from Tofts pharmacokinetic modeling of DCE MRI (**D**). Significant differences ( *p* < 0.05) compared to Ctrl tumors are indicated with a *.

The large vascular changes identified in AD tumors motivated a repeated experiment including tumors re-growing in the CR disease state. Figure 7A visualizes the development of CR tumors 5.4 ± 0.4 months after ADT by castration. Figure 7B shows the expression of serum PSA in blood samples from animals with Ctrl tumors, AD tumors, and CR tumors. The reduction in serum PSA from Ctrl to AD was significant (*p* = 0.032), whereas the rise in PSA from AD to CR was close to significant ( *p* = 0.085). The DCE MRI parameters best reflecting tumor vascularization, as identified from Figure 6, were calculated from the Ctrl, AD, and CR tumors. In Figure 7C, an increase in *AUC 1 min* and *AUC 2 min* from Ctrl tumors to AD tumors was again seen (1.8-fold increase, *p* = 0.011 and 1.7-fold increase, *p* = 0.025, respectively). The transition from AD tumors to CR tumors unveiled a further increase in tumor vascularization, as seen by the 1.4-fold increase ( *p* = 0.010) in *AUC 1 min* and a further 1.3-fold increase ( *p* < 0.001) in *AUC 2 min*. Also the Tofts parameters in Figure 7D showed similar pattern; a 1.3-fold increase (*p* = 0.012) in *K*^trans^ from Ctrl tumors to AD tumors, and a further 1.4-fold increase (*p* = 0.043) from AD tumors to CR tumors, whereas *k*_*ep*_ showed an insignificant change from Ctrl tumors to AD tumors, followed by a significant 1.6-fold increase (*p* = 0.007) from AD tumors to CR tumors.

## Discussion

In the present study the vascular responses to RT and ADT in experimental PC were characterized by qIHC and DCE MRI. Our key findings were the increased tumor vascularization following ADT, and the identification of a set of DCE MRI parameters non-invasively reflecting these vascular changes.

The xenograft used was originally derived from a patient’s primary, androgen-sensitive PC that later developed metastatic disease [14], thus being a clinically relevant model of advanced PC. This model enables investigations of the response to different interventions, in particular ADT, since this xenograft regresses after castration and shows stability before recurrent CR growth is evident, as also experimentally verified in this study (Figures 7A-B).

By qIHC an increased tumor vascularization was detected in AD tumors one month post-castration, compared to Ctrl tumors. This was reflected by the increased *VN*, *VD* and *VF*, although *VS* was smaller (Figure 3). Further, the vessels appeared to be highly functional despite their smaller size. This seems possible since the vessels were equally, or even more perfused as compared to Ctrl tumors, as reflected by unchanged *VPF* and the clear tendency of increased *PF* (Figure 3). Together, these findings are consistent with tumor neovascularization following ADT. We found a small, but insignificant, increase in *HF*. However, more interestingly the *DHV* was significantly shorter in AD tumors compared to in Ctrl tumors, which encouraged a further analysis of whether ADT had altered the ratio of acute versus chronic hypoxia independently of the total *HF*. For this purpose we estimated *AHS* (Figure 3K), revealing increased acute hypoxia, and thus less chronic hypoxia, in AD tumors compared to Ctrl tumors.

The mechanisms responsible for the ADT-induced changes in tumor vascularization and hypoxia are presumably caused by a complex interplay of different processes. First, a substantial increase in vessel formation may be expected during unperturbed growth from 8 mm (the diameter of tumors in Ctrl tumors) to 12 mm (the diameter of tumors at the time of castration). Following castration, volume reduction caused by death of androgen-sensitive, endothelial cells occur, likely resulting in vessel shrinkage. However, our results indicate a very high number of functional vessels with capability to transport blood, as reflected by the preserved tumor perfusion. Moreover, we observed an increased *VD* in AD tumors. One month after castration the tumor volume regressed by approximately a factor 3 (from a tumor volume of ≈ 864 mm^3^ (diameter of 12 mm) to a tumor volume of ≈ 256 mm^3^ (diameter of 8 mm)). A corresponding 3-fold increase in *VD* would thus be expected if the number of vessels remained identically as pre-ADT. However, the observed increase in *VD* was of a factor 5, and hence, a high number of vessels with smaller *VS* may not be explained by ADT-induced volume reductions alone. It has previously been shown that an immediate effect of ADT involves induction of acute hypoxia, including activation and stabilization of the hypoxia-inducible factor 1α (HIF-1α), and subsequent expression of HIF-1α target genes, such as the vascular endothelial growth factor (VEGF) [15-17], a key player involved in tumor neovascularization. Given that this occurs, formation of large numbers of small, new vessels may be expected. Such a neovascularization may explain the significant changes detected in vascular and perfusion-related qIHC parameters.

The *AHS* increased in AD tumors compared to in Ctrl tumors. Since perfusion qIHC parameters increased in AD tumors compared to in Ctrl tumors, it is not likely that the vessels are perfusion-limited or occluded, as is expected by the increased presence of acute hypoxia. Yet, small fluctuations in blood flow may result in temporary occlusions and transient hypoxia. However, such occlusions do not seem to severely have affected tumor vascularization and perfusion in AD tumors. In fact, it appears as if the vascular function in AD tumors is superior to in Ctrl tumors. It could be that the increase in acute hypoxia is followed by a reoxygenation of the tumor tissue. If extrapolating to the clinical situation, such a reoxygenation hypothesis may provide a possible explanation as to why neoadjuvant ADT combined with RT has been shown to improve treatment outcome and patient survival [2,3]. However, it has also been shown that reoxygenation after acute hypoxia contributes to increased genomic instability, and thus disease progression [18], including increased formation of metastasis [19,20].

The increased vascularization following ADT may also relate to development of a more aggressive disease, and hence, represent a step in the transformation into CR disease. Clinical studies in PC have previously connected microvessel density to poor survival [21-23]. When we repeated the experiment and extended it to also include CR tumors, where the serum PSA levels increased and renewed growth appeared, we detected an even further increased tumor vascularization compared to both Ctrl tumors and AD tumors (Figure 7C and D). Highly vascularized tumors are usually well oxygenated and sensitive to radiation. These tumors may, paradoxically, have enhanced activation of angiogenic factors normally found in hypoxic, radiation resistant tumors and in tumors associated with a poor prognosis [24]. Both for breast cancer and colorectal cancer correlations between enhanced expression of angiogenic factors and poorer prognosis and shorter disease-free survival have been shown [25,26]. Moreover, highly angiogenic tumors are more likely to develop metastatic disease as the probability of secreting tumor cells into the circulation is higher [27].

Human studies characterizing vascular effects following ADT are limited. Animal studies have shown that immediate responses to ADT involve blood flow reductions and vascular degenerations [28-31]. The recent study by Godoy *et al.*[6] shows vascular recovery after ADT, as demonstrated by increased microvessel density and vascular lumen diameter. The difference in the study by Godoy *et al.* to previous studies is that they not only characterized the immediate effect of ADT on tumor vasculature, but prolonged their post-ADT investigation period. Thus, these results indicate that ADT induces an immediate, transient degeneration of tumor vascularization before androgen-sensitive, endothelial cells develop alternative mechanisms to reproduce the vascular network in the absence of androgens. A recent DCE MRI study in PC patients oppositely demonstrated decreased *K*^trans^ after 1 and 3 months of ADT [32]. One explanation for the different results may be related to the timing of the measurements. The clinical study may comply with the preclinical results of initial vascular degenerations. Our results, obtained 1 month post-ADT, anticipated to correspond to about 5 months in humans after accounting for the five times faster metabolism in mice, may reflect a later disease stage with vascular recovery, and comply with the results by Godoy *et al.*[6]. The challenges with translating time-dependent treatment effects in xenografts to humans indicate that studies characterizing the long-term effects of ADT on tumor vascularization should be further investigated in the clinical setting. One approach could be to perform DCE MRI also at later disease stages after ADT, preferably as repeated measurements in the same patients.

The results from qIHC following RT showed results being consistent with previous findings. RT did not result in substantial effects on *VD* and *VF* 24 h post-irradiation, only an insignificant increase in vessel size *VS* was found. The increased *VS* may relate to radiation-induced dilatation of tumor capillaries, as has been previously shown [24,33]. Further, RT resulted in a significant decrease in *PF,* as also shown by others [34]; this is likely attributed to the formation of radiation-induced edemas and capillary injuries. Successful RT results in irreversible DNA damages, which are in accordance with the increased *NF* in the RT tumors. Moreover, it is also previously shown that vascular alterations after RT can be detected by DCE MRI [35].

Vascular qIHC parameters, like *VD* and *VF*, correlated strongly to *AUC* values from the initial time-points (Figure 6). Moreover, *AUC* values were negatively correlated to *DHV*. This may be related to the higher fraction of viable cells, as reflected by the *VF70V* parameter, close to the vessels in tumors with short *DHV*. Applying the Tofts kinetic model, the *k*_*ep*_ parameter improved the correlation to *VS* compared to using *AUC* values. *VD* was also positively correlated to the Tofts parameters, albeit less than the *AUC* values. In summary, the correlations between DCE MRI parameters and vascular qIHC parameters showed that *AUC* values reflect *VD* and *VF* strongly, whereas estimation of *VS* from DCE MRI requires the use of the Tofts kinetic model. Since parameters from the Tofts model previously have shown to be indirectly related to tumor hypoxia [7,36], the poor correlations between *HF* and all DCE MRI parameters were surprising. It should be noted that the *HF* was not found to correlate to other qIHC parameters either, nor differentiate treatment groups in the *t*-test. One possible reason may be that the analyzed tumors, with a few exceptions, showed relatively similar *HF*, and hence, the spread in the data were too low to resolve any such correlation. By estimating *AHS* (Figure 3K), substantially higher and significantly positive correlations to several DCE MRI parameters were found (Figure 6).

The *NF* was strongly correlated with the unfit fraction from the Tofts model, i.e. the voxels with low contrast enhancement. Bradley *et al.*[37] and Galbraith *et al.*[38], as well as our own previous study [10], have revealed similar correlations, providing support for the unfit fraction as a non-invasive biomarker of tumor necrosis.

In this study, the potential of DCE MRI to provide non-invasive biomarkers of tumor vascularization was assessed. After the recognition of the importance of the condition of the tumor vasculature, including tumor hypoxia, for treatment response and disease outcome in oncology [39,40], several functional imaging modalities have been investigated for their ability to provide such non-invasive biomarkers. In addition to DCE MRI and diffusion-weighted (DW) MRI, positron emission tomography (PET), dynamic computed tomography (CT), and ultrasound Doppler imaging, have been proposed as promising modalities. The benefits with MRI include that it is a wide-spread, clinically established modality, also in advanced PC, and hence, any new sequences, such as DCE MRI, may easily be added. Moreover, compared to PET and CT, MRI benefits from the avoidance of ionizing radiation exposure, as well as being more cost-effective.

In conclusion, DCE MRI non-invasively detected an increased and highly functional vascular network in experimental prostate tumors after ADT, which was confirmed by qIHC. The findings encourage further studies into whether these vascular changes are beneficial for combined RT, or if treatment with anti-angiogenic therapy or vascular disrupting agents may be a strategy to improve the therapeutic efficacy of ADT in advanced PC.

## Competing interests

The authors report no competing interests.

## Authors’ contributions

KR participated in study design, carried out the animal experiments, MRI data acquisition and analysis, and wrote the manuscript. LTGM performed quantitative analysis of immunohistochemical stains and participated in writing of the manuscript. AJvdK and JB were responsible for fluorescent immunohistochemical staining of tissue sections. HL participated in data discussion and revision of the manuscript. AHR, LM, and DRO participated in study design, data discussion and revision of the manuscript. All authors read and approved the final manuscript.
